# Health-Related Quality of Life in Kindergarten Children with Developmental Language Disorder: Child–Mother Agreement

**DOI:** 10.3390/bs13121017

**Published:** 2023-12-18

**Authors:** Maria Boukouvala, Thomas Hyphantis, Iouliani Koullourou, Alexandra Tzotzi, Andromachi Mitropoulou, Christos Mantas, Petros Petrikis, Aspasia Serdari, Vassiliki Siafaka, Konstantinos Kotsis

**Affiliations:** 1 Department of Psychiatry, Faculty of Medicine, School of Health Sciences, University of Ioannina, 45 110 Ioannina, Greece; m.boukouvala@uoi.gr (M.B.); tyfantis@uoi.gr (T.H.); j.koulourou@uoi.gr (I.K.); alexandratzotzi@gmail.com (A.T.); mitropoulouandro@hotmail.com (A.M.); ppetrikis@uoi.gr (P.P.); 2 Department of Child and Adolescent Psychiatry, Medical School, Democritus University of Thrace, 68 100 Alexandroupolis, Greece; aserntar@med.duth.gr; 3 Department of Speech & Language Therapy, School of Health Sciences, University of Ioannina, 45 500 Ioannina, Greece; siafaka@uoi.gr

**Keywords:** developmental language disorder, HRQoL, child–parent agreement, kindergarten, preschool

## Abstract

Language disorders are associated with difficulties in various aspects of life, such as academic and social functioning, resulting in impaired health-related quality of life (HRQoL). Most studies use a parent proxy method to assess HRQoL. Since HRQoL refers to the subjective experience of an individual, it is necessary to assess children’s perspectives along with their mothers’. The aim of the current study is to explore HRQoL rating agreement between children and their mothers, since the literature on other conditions suggests that discrepancies seem to reflect their different perspectives. Thus, 53 Greek-speaking children diagnosed with DLD attending kindergarten and their mothers completed, respectively, self-report and parent proxy PedsQL^TM^ questionnaires. Mothers reported significantly better HRQoL than their children with developmental language disorder (DLD) in all HRQoL domains (*p* < 0.001). Poor agreement was revealed after comparing the scores from both responders, both in abstract domains, such as emotional functioning, as well as in more observable ones, such as physical health (ICC ranged from −0.05 to 0.07). Bland–Altman plots also showed poor agreement on HRQoL. Our results expand on the already known, from other conditions, importance of evaluating children’s subjective experience of their HRQoL in kindergarten children with DLD. A multi-informant approach is ideal, and clinicians should prioritize children’s view about their lives even when they are kindergarten-age. This approach could inform interventions focusing not only on language skills but also on other areas where it is necessary, depending on the child’s subjective experience combined with the maternal perspective.

## 1. Introduction

Parents have been considered to have knowledge about the feelings and thoughts of their children, and therefore to be able to represent them with accuracy in health-related issues. However, this conception has been challenged since research has shown that the views of children and their parents may differ [1,2,3]. Given this, the primary aim of the current study is to explore the agreement in terms of health-related quality of life (HRQoL) between mothers and kindergarten-age children diagnosed with developmental language disorder (DLD).

DLD is a quite common developmental disorder diagnosed in the preschool years. It refers to low language ability in the absence of biomedical conditions that could explain the deficit [4,5]. According to the literature, 6–8% of preschool children have DLD [6]. Children with DLD have significant difficulties in learning, understanding, and using spoken language. Moreover, their educational performance may be affected (reading disabilities, spelling problems, math) [7]. Different terms (specific language impairment, language delay, developmental language disorder, and developmental dysphasia) have been used for language disorders, but currently the consensus is to use the term DLD and this term will be used in this study hereafter [4,5,8]. It is known that DLD has been associated with difficulties in various aspects of children’s life, such as psychosocial functioning [7,9,10,11]. The literature suggests that children with DLD face difficulties with relationships, emotions, achievement, independence, and support while adolescents are more likely to experience anxiety and depression [7,12,13,14]. Moreover, adults with a history of DLD are twice as likely to go over a year without employment as other adults [15]. An assessment of the impact of language disorders on children’s functioning might be useful, as clinicians could address this impact during the intervention process, along with interventions focusing on language skills. A recent study [16] reported that more than half of children with poor language had declining trajectories in HRQoL from 4 to 13 years old, indicating that interventions should also address the functional impact of low language.

HRQol in Developmental Language Disorder

Children’s functioning can be assessed by measuring their HRQoL, which is a term that encompasses an individual’s perspective on how a disorder impacts their life [17]. Specifically, HRQoL is defined as ‘‘those aspects of self-perceived well-being that are related to or affected by the presence of disease or treatment’’ [18]. Moreover, HRQoL is considered a subdomain of quality of life, which includes health-related domains of life [2]. Simply, HRQoL can be defined as a useful indicator of overall health because it captures information on the physical and mental health status of individuals, and on the impact of health status on quality of life [19]. Many studies [16,20,21] have explored HRQoL in children with language problems. According to the literature, a mild presentation of DLD (the term used in this study was specific language impairment) in children aged 8–11 years old has an impact on sleep and speech but not on overall HRQoL score, according to their own reports [22]. However, when it comes to more severe presentations of the disorder, HRQoL was affected, especially in terms of social and physical functioning, according to children’s (5–16 years old) and parents’ views [23]. Moreover, parents of children aged 8–18 years old with DLD recorded significantly lower HRQoL scores on all dimensions compared with the parents of their typical peers [24]. In a prospective study [14], children with DLD had lower parent-reported HRQoL than their typical peers at 9 years old, but these differences were not associated with DLD severity, except for school functioning.

There is an ongoing debate on the most appropriate respondent for the assessment of children’s HRQoL in the health outcome literature [25,26]. HRQoL captures the subjective experience of an individual related to their own impairment (among other things), and therefore it is important to ask children for their own self-report, rather than taking into consideration only parent reports [27]. Children with communication disorders may find it difficult to accurately report their own experience concerning HRQoL [26,28,29]. However, there are studies suggesting that five-year-old children or even younger children may be able to express their subjective view on HRQoL and well-being [29,30]. Above all, in medical care and child research, it is highly recognized that children’s self-reporting of HRQoL is of high value [31]. Parents may also provide useful and valid information on some aspects of their children’s life that are more observable, but this might be less valid when evaluating more abstract issues such as emotional aspects [3,26,32]. Children and parents may evaluate HRQoL based on different factors, e.g., parents may worry about future school functioning, which might not be the case for a child in kindergarten with language disorder. Therefore, parent reports cannot substitute child reports, a problem known as the proxy problem [33]. In this context, the importance of obtaining children’s self-reports about their functioning, well-being, and health is recognized in clinical and research settings; therefore, a combination of child self-reported and parent-proxy-reported HRQoL may provide more comprehensive information about the impact of a condition on a child’s life [31].

Agreement studies in developmental disorders are limited. In a recent study, Ottosson et al. [34] found that 6-year-old children with DLD reported impairment mainly in school functioning, whereas parents reported no impairment in the children’s quality of life. It is worth noting that this was a comparison study, but no agreement analysis was used. In children under 6 years old with developmental coordination disorder (DCD), authors [35] found no differences in reported HRQoL between children and their parents. Furthermore, in a study [36] with school-age children suffering from Tourette syndrome, significant agreement between children’s and their parents’ reports was found on all HRQoL dimensions and psychosocial functioning domains of PedsQL. Finally, a systematic review [1] including medical and developmental conditions revealed moderate and poor levels of child–parent agreement; higher agreement was found on observable than on non-observable domains.

The literature is sparse in terms of parent-reported HRQoL in children diagnosed with DLD and non-existent regarding children’s perspectives on HRQoL. However, the sample in an Australian study included children with a starting age of 5 years, but the mean age was 8.74 [21]. It is important to obtain children’s perspective on their HRQoL since there is evidence that, when provided with age-appropriate information, they can communicate their health needs [37,38].

This also applies to children and adolescents with language problems, as research has shown that they are aware of their communication difficulties [39]. HRQoL studies have the potential to aid in identifying the children’s needs and guide interventions for children with DLD [24]. Various studies have used HRQoL measures in children and adolescents with language problems, since it is important for their experiences to be acknowledged, as has been highlighted in the United Nations Convention of the Rights of the Child [20,40,41,42]. To the best of our knowledge there are no studies exploring the level of child–parent agreement on HRQoL in kindergarten children with DLD. Information about HRQoL provided by both children and parents might be used to inform clinical decisions when providing interventions or counselling to parent–child pairs [43]. Given this gap, the aim of this study was to explore how kindergarten children diagnosed with DLD and their mothers report the children’s HRQoL and if their ratings agree or disagree. We hypothesize, based on previous research on other conditions, [2,28] that for more abstract concepts, such as psychosocial domains, the level of agreement will be low, whereas for more observable aspects, such as physical health, the level will be high. Findings from studies like the current one could inform interventions considering both children’s and parents’ perspectives. Therefore, interventions could target aspects of HRQoL (e.g., social and emotional functioning) affected by language difficulties and improve children’s well-being.

## 2. Materials and Methods

### 2.1. Participants

Participants included 53 Greek-speaking children attending kindergarten (in Greece, 5–6 years old) and their mothers. They were recruited from a community child and adolescent mental health service (CAMHS) at a tertiary university hospital between January 2021 and March 2022. The mean age of the children was 5.36 ± 0.48 (range 5–6) and there were 30 boys and 23 girls. All of the children were enrolled in kindergarten, as preschool education is mandatory in Greece. Additionally, the sample exclusively included mothers, as they are typically considered the primary caregivers [44], with a mean age of 38.51 ± 5.2 years.

In Greece, the common pathway for the evaluation of children with language difficulties is to request a diagnostic assessment to be completed at a CAMHS. The intervention could be completed in the public sector or privately. All children diagnosed with DLD in our CAMHS during the study period, and their parents, were asked to participate in the study. None of the parents or children refused to participate in the study. All children in our sample underwent clinical assessment by a speech and language therapist and then by a child and adolescent psychiatrist to exclude other developmental disorders such as autism spectrum disorder (ASD) or intellectual disability (ID). Diagnosis was made by clinical evaluation, and eligibility criteria for participation in the study were (a) diagnosis of developmental language disorder independently of severity; (b) not following a speech—language intervention; (c) being native speakers of Greek; (d) presenting only expressive and not receptive language difficulties; and (e) not suffering from any medical condition (that could affect HRQoL) or having any mental or other developmental disorder. All mothers and children were informed about the purposes of the study and confidentiality verbally and in writing by the speech and language therapist; mothers were requested to sign a consent form while the children provided their consent verbally. Mothers completed the parental forms in the waiting room, while the children independently filled out the questionnaire administered by the speech and language therapist after their child psychiatry evaluation, without the presence of their mothers.

The study was conducted according to the guidelines of the Declaration of Helsinki and ethical approval was obtained from the ethical committee of our institution (University Hospital of Ioannina, Reference number: 989—21December 2020).

### 2.2. Measures

In this study, Pediatric Quality of Life Inventory (PedsQL^TM^) 4.0 Generic Core Module self- and parent-proxy-reported HRQoL was used [29,45,46,47]. Specifically, the young child self-report and parent-proxy version for ages 5–7 were used. The young child self-report version employs a simplified 3-point Likert scale going from ‘not at all’ to ‘a lot’ with smiley faces to aid children complete the rating task. Specifically, the three options are not at all (indicated as 0), sometimes (indicated as 2), and a lot (indicated as 4). The parent proxy version employs a 5-point Likert scale (from 0 to 4 as in the child version) going from “never” (indicated as 0) to “almost always” (indicated as 4). Both versions comprise 23 items distributed across 4 dimensions: physical functioning (8 items), emotional functioning (5 items), social functioning (5 items), and school functioning (5 items). Each statement asks the child or the parent: “How much of a problem has this been for you/your child?”. Examples for the child and parent versions for each dimension respectively are: physical functioning: “it is hard for me to do sports activity or exercise/participating in sports activity or exercise”; emotional functioning: “I feel sad or blue/feeling sad or blue”; social functioning: “Other kids tease me/getting teased by other children”; and school functioning: “It’s hard to pay attention in class/paying attention in class”. The scales generate two summary scores: a psychosocial health summary score (the sum of the emotional, social, and school functioning dimensions) and a physical health summary score which is identical to the physical functioning dimension. Finally, a total score is calculated from the sum of all of the items over the number of items answered (this accounts for missing data). If more than 50% of the items in the scale are missing, the scale score is not computed. All items (both in the child and the parent versions) are reverse-scored and linearly transformed to a 0–100 scale (e.g., 0 = 100, 1 = 75, 2 = 50, 3 = 15, 4 = 0) where higher scores indicate better HRQoL.

### 2.3. Statistical Analysis

The Statistical Package for Social Sciences v28 for MacOS and the Blandr Jamovi Module were used [48,49]. Mean values for continuous variables and percentages for categorical variables were used. We assessed differences in self-report and parent-proxy HRQoL reports separately for each dimension, summary scores, and total score using the non-parametric Wilcoxon rank test. The statistical significance of the *p*-value was set to 0.008, after Bonferroni correction. The internal reliability (Cronbach’s alpha coefficients) of both versions (child and parent) of the PedsQL^TM^ 4.0 was calculated. The level of agreement between child self-reports and parent proxy-reports was analyzed using intraclass correlation coefficient (ICC) absolute agreement. ICC values less than 0.5, between 0.5 and 0.75, between 0.75 and 0.9, and greater than 0.90 are indicative of poor, moderate, good, and excellent reliability, respectively [50]. Negative ICC values indicate that the difference between subjects is larger than the difference within subjects. Individual child–parent agreement was also evaluated by visual inspection of the Bland–Altman plots with the *y*-axis representing differences in child–parent agreement plotted against their means (plotted on the *x*-axis). An advantage of the Bland–Altman plot over ICC is that it gives an indication of the discrepancy from equality and also reveals the range of the values. The limits of agreement were computed, and perfect agreement between a child and a parent entails that the discrepancy score is equal to zero. The plot can be evaluated according to the dispersion of the dots. If the dots are located close to the mean bias line and the scattering of the dots is diminished, then there is high agreement. The more dispersed the dots are, the poorer the agreement. A sample size of approximately 50 patients is required to provide a reasonable number of dots in a Bland–Altman plot to estimate the limits of agreement [51].

## 3. Results

### 3.1. PedsQL Reliability

Cronbach’s alpha for the parent version in this study was 0.83, indicating a good reliability level, whereas the alpha coefficient for the child version was 0.71, indicating that the scale is acceptable.

### 3.2. Child Self-Reported and Parent-Reported HRQoL

Table 1 shows the mean scores in each dimension, summary scores, and total score for children and mothers’ reports. Children recorded the highest score in the school functioning dimension (78.7 ± 13.6) followed by physical (73.1 ± 13.8) and social functioning (72.6 ± 22.2). The lowest score on the HRQoL was recorded by the children in the emotional dimension (64.9 ± 19.0). A similar pattern was observed in maternal reports. Mothers evaluated their children’s physical functioning as highest (87.6 ± 13.8), followed by school (87.0 ± 13.1) and social (85.6 ± 16.1) functioning. Mothers reported that the emotional functioning of their children was lower compared with other dimensions of HRQoL.

In all dimensions, summary scores, and total score, mothers rated their child’s HRQoL statistically significantly (*p* < 0.001) higher compared with the subjective experience of their children. This finding indicates that mothers probably overestimated the HRQoL of the children.

### 3.3. Level of Child–Parent Agreement

Child–parent agreement was very poor (ICC < 0.40) for all aspects of HRQoL. Physical, social, and school functioning as well as physical health had ICC values of 0.07, 0.07, 0.05, and 0.07, respectively, indicating very low child–parent agreement. Emotional functioning, psychosocial health, and total score had negative ICC values. Therefore, on these dimensions, mothers and their children differ from each other, on average, more than each child differs from the other children (Table 2).

**Figure 1:**
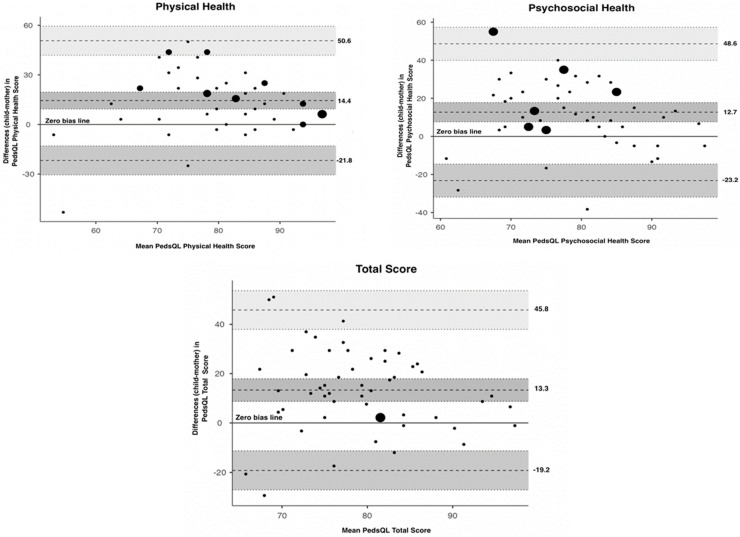
Bland–Altman plots for child–mother agreement on the PedsQL^TM^ physical health, psychosocial health, and total scores. The *y*-axis represents differences in child–parent agreement plotted against their mean (plotted on the *x*-axis). Perfect agreement between a child and a parent entails that the discrepancy score is equal to zero and this is plotted as the zero-bias line. The upper and lower limits of agreement are plotted as dashed lines.

To further examine child–parent agreement on the physical and psychosocial scores as well as the total score for the PedsQL^TM^, Bland–Altman plots (Figure 1) were plotted. Child–parent agreement differences are indicated by dots, while the size of the dots increases with increasing numbers of child–parent pairs with the same difference in score.

The observed (lower and upper) limits of agreement (Table 3) for physical health were −21.8 and 50.6, for psychosocial health −23.2 and 48.6, and for total PedsQL^TM^ score −19.2 and 45.8. The mean difference in the physical score was 14.4, in the psychosocial score was 12.7 and in the total score was 13.3, indicating that mothers rate their children’s HRQoL higher than their children for the above values (on average). Visual inspection shows a wide dispersion of the dots in all three plots, indicating poor agreement. Moreover, we can assume that the bias is significant since the line of equality is not within the confidence interval of the mean difference. Similar poor agreement was also observed for each dimension of the PedsQL^TM^. 

Overall, the observed poor agreement and the different ratings for HRQoL between children and their mothers highlights the importance of considering both perspectives in the intervention process and trying to help mothers to understand more clearly the perceptions of their children regarding their own HRQoL.

## 4. Discussion

The findings in the present study reveal that there is poor agreement between children and mothers in their assessments of HRQoL levels. In general, mothers reported better HRQoL than their children, which is not entirely consistent with the literature. A recent review [1] concluded that, in general, parents tend to perceive their children as having more difficulties than the children themselves believe they have, both in terms of physical and psychosocial health issues. We assume that the parents in our sample may have underestimated the impact of DLD on the lives of their children, because DLD is often considered a hidden impairment [7]. This might be the case for studies with community samples as well as in our study. This is because DLD is considered a developmental condition and not a disease, and its impact might not be as pronounced as in other developmental disorders like ASD or in medical conditions such as epilepsy [2].

Furthermore, within individual child–parent pairs, the level of agreement varied significantly, as indicated by the wide intervals between the limits of agreement for both physical and psychosocial health, as well as HRQoL total score. To the best of our knowledge, this is the first study in the literature to explore the level of agreement in HRQoL exclusively within a sample of kindergarten children with DLD. Our results differ from the previous literature, as we found poor agreement in all domains of HRQoL.

- HRQoL agreement studies in developmental disorders

Previous studies [35,36] exploring child–parent agreement focused on children with other developmental disorders (DCD, Tourette syndrome), not DLD. A meta-analysis of the HRQoL of children with ADHD and their parents concluded that ratings did not differ significantly, but there was substantial variability in agreement across individual studies and with different HRQoL measures [52,53]. Poor agreement was found in a study involving children with ASD [54], potentially due to the communication difficulties autistic children often face. The same applies to adolescents with ASD in a study [55] that found low agreement for self-perception, autonomy, and parent relation. In Greek children with specific learning disorders poor agreement was also found [56]. This variation may be attributed to differences in the disorders themselves and the age spectrum studied. For example, children with DCD and Tourette syndrome do not experience language/communication difficulties. As a result, they may be able to self-report their own HRQoL experiences, leading to higher agreement between their reports and those of their parents. Therefore, the absence of language difficulties might help parents to understand their children’s HRQoL reports better. Consequently, agreement among parent and child HRQoL reports might be higher in conditions having no symptoms of language difficulties. Therefore, the presence of language difficulties combined with the young age of our sample may explain the difference in agreement compared with other studies involving neurodevelopmental disorders such as DCD and Tourette’s. There are also studies reporting mixed results. In a study of adolescents with intellectual disability, a low correlation was found on the scales of physical and emotional health, while a moderate correlation was obtained on the school functioning subscale, the psychosocial summary scale, the social subscale, and the total scale [57].

- DLD and HRQoL agreement

Literature suggests [2,3,28] that the agreement between parents and children in more abstract domains of HRQoL is low while it might be higher in more observable aspects such as physical health. However, this was not the case in our study, even if agreement was found to be poorer in abstract domains compared with more observable ones. The question is why this discordance between mother proxy reports and child self-reports exists in all domains of HRQoL in kindergarten children with DLD and why mothers significantly overestimate their children’s HRQoL. One possible reason, especially on abstract domains (e.g., emotional functioning) may be that children of that young age might not be able to convey their feelings to their parents about the impact of their language difficulties on their lives. Moreover, due to their language difficulties they might not be able to express their feelings precisely. We cannot rule out the possibility that a child’s capacity to understand the items in the questionnaire may have been compromised by the presence of DLD; yet, the questionnaire was administered with a speech therapist in the evaluation room, available for clarifications. Results in the literature, involving children diagnosed with various health conditions, have been inconsistent in relation to age in the discordance between child and parent agreement in HRQoL measures. There are studies showing that the agreement between child and parent is significantly lower for the youngest children of the family compared with the oldest ones and studies stating that the agreement is lower in adolescents compared with young children [58,59,60]. In Greek children (mainly from the general population), poor to moderate agreement has been found, especially among younger age groups. This has not been the case for children attending kindergarten, but this (poor to moderate agreement) was observed among 8- and 9-year-olds [61]. Considering this, the even-poorer agreement in our sample, which consists of kindergarten children with language difficulties, seems rather reasonable. Moreover, language difficulties per se in our DLD sample may exacerbate the limited ability of kindergarten children to report their emotional status. Therefore, mothers may be unaware of these non-observable experiences of their children as well as their non-expressed feelings. This could also apply to social functioning because mothers might not be able to evaluate the quality of the relationship or the frustration that a child with DLD may experience when communicating with their peers. Mothers may also rely on social comparisons to inform their decisions about the quality of life of their children. Since there is variability in children’s language at that age, and children with DLD, especially of mild severity, may be closer to the norm, mothers may not be able to accurately determine HRQoL. A similar reason may account for the physical domain, which is a quite surprising result given the fact that it is an observable domain. Children as young as 4–5 years old with DLD may not find it easy to report their everyday difficulties in physical health to their mothers. Moreover, as HRQoL is a measure of subjective experience, parents may not report their observations but how difficult they believe a task for their children is, which is not quite observable. Furthermore, many parents might not know the developmental motor milestones (e.g., throwing a ball overarm, unbuttons buttons, dressing and undressing without assistance) [62]. Of course, these physical difficulties are not the consequence of a language disorder, but the language disorder could be the reason for the limited ability of children to inform their parents about physical difficulties. In general, an explanation of why agreement may be poor due to language difficulties might lie in the poor language skills of children with DLD. It is known that children with DLD have more reduced vocabularies than their peers and have difficulties mapping labels to objects, and low language abilities may not promote the representation of emotional experiences [63,64,65]. It is possible for all these reasons that children may not express their thoughts and feelings accurately to their parents. Therefore, parents may not understand that they struggle and overestimate their HRQoL.

The literature is sparse concerning HRQoL in kindergarten children with DLD; we could not identify studies using self-reported measures solely in kindergarten children with DLD to compare our results. The lowest score for HRQoL in our sample was reported in emotional functioning and this might reflect the impact of the pandemic [66,67], since our study was conducted during the COVID-19 era.

### 4.1. Limitations

The present study has several limitations. First, the cross-sectional nature of the study limits our ability to catch changes in HRQoL; thus, we cannot argue that the results are directly a consequence of DLD. Therefore, longitudinal studies are needed to understand fully whether the agreement persists in other developmental periods such as school age and adolescence. Second, we did not account for the severity of DLD; therefore, we are not sure that the agreement would be the same in mild and severe presentations of the disorder. In that context, future studies should weigh participants according to their severity. Moreover, despite the existence of Greek PedsQL^TM^ data, the lack of a control group limits the interpretation of whether children in our sample with DLD have impaired HRQoL. Therefore, future studies with a control group could provide more accurate information about the HRQoL of children with DLD. Additionally, the use of a generic HRQoL measure instead of a specific one for DLD raises the question of missing important dimensions of psychological functioning specific to children with DLD. Moreover, generic measures cannot assess outcomes adequately in DLD [20]. There are some specific measures for language disorders; however, they are not as extensively used and some of them are not validated in the pediatric population. There is little convergence that exists for a gold-standard HRQoL measure in DLD. As DLD may influence the capacity of children to accurately report their HRQoL, specific measures may overcome this barrier and give more appropriate information in future studies [20]. Furthermore, we collected data only from mothers, so we missed fathers’ perceptions; future research in HRQoL agreement measures should also include them. One more limitation is the lack of standardized assessment measures (for the DLD diagnosis as well as for exclusion conditions), which limits the replicability of our results. Finally, the sample size may be considered small given the prevalence of DLD, even though it is adequate for statistical analysis. Moreover, in terms of representativeness, even if our CAMHS is the only public service in the region, many parents who can afford evaluation may seek it from private practice. However, the use for the first time of the PedsQL^TM^ solely in kindergarten children with DLD as well as the clinical assessment (by the SLP and child psychiatrists to exclude comorbidity) of all children represent the strengths of our study. Additionally, children filled out the questionnaire without their mothers being present; therefore, this helped to eliminate the potential for children and mothers to collude and give more moderated responses.

### 4.2. Clinical Implications

We acknowledge that in clinical settings there is a need for a multi-informant approach in the assessment of children’s health issues; thus, parent (and teacher) proxy-reports are essential for a comprehensive evaluation. We would like to stress, however, the importance of prioritizing children’s right to express their views in all matters affecting them. Our findings show that mothers, on average, overestimate their children’s HRQoL, but on the individual level there are dyads where the opposite can be observed. Therefore, we highlight the fact that both perspectives should be considered, as we believe that both are valid and broaden the view of a child’s well-being.

## 5. Conclusions

Mothers of kindergarten children with DLD rated their children’s HRQoL differently and significantly better compared with the rating provided by the children themselves. Our results suggest the importance of evaluating children’s reports and not depending solely on the mother’s perspective. While the poor agreement raises concerns about the accuracy of mothers’ ratings, we cannot argue that either mothers’ or children’s reported HRQoL is more accurate than the other; they may simply reflect different perspectives. Indeed, a systematic review [1] concludes that discrepancies between child and parent reports reflect their different perspectives rather than an inaccuracy or a bias. This finding should be used by clinicians who should use both parent and child measures for decision-making in pediatric settings. The question is not whose perspective is right, but where each perspective may advance the intervention process to ensure the well-being of the child with DLD. In clinical settings, obtaining both perspectives may guide a more accurate conceptualization of the child’s overall functioning that might inform a more comprehensive intervention plan. For example, clinicians may address, during the intervention process, children’s emotional issues that are not captured by mothers and evaluate HRQoL as an outcome of their interventions. Moreover, mothers may participate in a consultation process to better understand their children’s perspectives. Furthermore, in educational settings, educators might be alert for issues other than language difficulties (e.g., social or school functioning) and inform parents for them to have a more comprehensive view of their child. On the other hand, parents may inform educators (and school mental health professionals) about their child’s own experience of functioning and therefore assist them in helping the child in the school setting; e.g., by enhancing their social abilities. This practice may assist in providing comprehensive care to children with DLD and ensuring that they receive health and educational services from which they would most benefit. Moreover, researchers should focus on factors that may contribute to the discrepancy between parents’ and children’s perspectives. Finally, studies should include children with clinical presentations of different severity, considering that DLD is an under-researched condition. In conclusion, our findings emphasize the importance of obtaining children’s views regarding their own experience, even if they are of preschool age. Early intervention not only in language skills but in general in various aspects of HRQoL may minimize the impact of DLD. Expanding DLD research in all age groups and exploring its impact in various respects may help DLD stop being an underserved and under-researched disorder [7].

## Figures and Tables

**Table 1:** Mean and SD for all HRQoL domains on the child self-reported and parent-proxy-reported PedsQL^TM^ 4.0.

HRQoL Domains	Child Self-Report	Parent Proxy Report	*p*
	Mean ± SD (N = 53)		
Physical Functioning	73.1 ± 13.8	87.6 ± 13.8	<0.001
Emotional Functioning	64.9 ± 19.0	81.8 ± 14.7	<0.001
Social Functioning	72.6 ± 22.2	85.6 ± 16.1	<0.001
School Functioning	78.7 ± 13.6	87.0 ± 13.1	<0.001
Physical Health	73.1 ± 13.8	87.6 ± 13.8	<0.001
Psychosocial Health	72.1 ± 13.8	84.8 ± 11.1	<0.001
Total Score	72.4 ± 12.2	85.7 ± 10.3	<0.001

**Table 2:** Agreement between child self-report and parent proxy report on all HRQoL domains on the PedsQL^TM^ 4.0.

HRQoL Domains	ICC
Physical Functioning	0.07
Emotional Functioning	−0.05
Social Functioning	0.07
School Functioning	0.05
Physical Health	0.07
Psychosocial Health	−0.05
Total Score	−0.05

ICC: Intraclass correlation coefficient.

**Table 3:** Bland–Altman Analysis for PedsQL^TM^ Physical Health, Psychosocial Health, and Total Score.

HRQoL Domains	Limit of Agreement		
	Bias Estimate (95% CI)	Lower (95% CI)	Upper (95% CI)
Physical Health	14.4 (9.3–19.5)	−21.8 (−30.5–−13.0)	50.6 (41.9–59.4)
Psychosocial Health	12.7 (7.6–17.8)	−23.2 (−31.9–−14.5)	48.6 (39.9–57.3)
Total Score	13.3 (8.7–17.9)	−19.2 (−27.1–−11.3)	45.8 (37.9–53.7)

CI: Confidence Intervals.

## Data Availability

Data are available from the authors upon request.
